# Antimicrobial Drug Prescribing for Pneumonia in Ambulatory Care

**DOI:** 10.3201/eid1103.040819

**Published:** 2005-03

**Authors:** Conan MacDougall, B. Joseph Guglielmo, Judy Maselli, Ralph Gonzales

**Affiliations:** * University of California School of Pharmacy, San Francisco, California, USA; † University of California School of Medicine, San Francisco, California

**Keywords:** research, pharmacoepidemiology, pneumonia, bacterial, managed care programs, fluoroquinolones, drug resistance, microbial

## Abstract

To determine patterns and predictors of antimicrobial drug use for outpatients with community-acquired pneumonia, we examined office visit and pharmacy claims data of 4 large third-party payer organizations from 2000 to 2002. After patients with coexisting conditions were excluded, 4,538 patients were studied. Despite lack of coexisting conditions, fluoroquinolone use was commonly observed and increased significantly (p < 0.001) from 2000 to 2002 (24%–39%), while macrolide use decreased (55%–44%). Increased age correlated with increased fluoroquinolone use: 18–44 years (22%), 45–64 years (33%), and >65 years (40%) (p < 0.001). Increased use of fluoroquinolones occurred in healthy young and old patients alike, which suggests a lack of selectivity in reserving fluoroquinolones for higher risk patients. Clear and consistent guidelines are needed to address the role of fluoroquinolones in treatment of outpatient community-acquired pneumonia.

## Abstract 2

Higher levels of fluoroquinolone use were associated with increasing age and later study year.

Community-acquired pneumonia (CAP) is a leading cause of death due to infection in the United States and a primary indication for antimicrobial drug use in inpatient and outpatient settings. The fluoroquinolone class of antimicrobial agents has become increasingly popular for the management of CAP because of coverage of common CAP pathogens, toleration by patients, and excellent oral absorption (*1*). “Respiratory” fluoroquinolones, such as levofloxacin, gatifloxacin, moxifloxacin, and gemifloxacin, have activity against most strains of drug-resistant *Streptococcus pneumoniae* (DRSP) (*2*). Concerns for infection due to DRSP may drive fluoroquinolone use because providers fear that traditional CAP regimens will fail (*3*,*4*). However, fluoroquinolone resistance, while generally low, appears to be increasing in *S. pneumoniae* (*5**–**8*). Therapeutic failures have been reported in patients infected with fluoroquinolone-resistant organisms treated with levofloxacin (*9*,*10*). Although data from community settings are lacking, resistance to fluoroquinolones is also increasing among gram-negative organisms in hospital settings (*11*,*12*). Increased use of fluoroquinolones for outpatient respiratory tract infections may lead to increased resistance rates among community-acquired gram-negative organisms. Fluoroquinolones may promote colonization and infection with methicillin-resistant *Staphylococcus aureus* (MRSA) (*13*–*15*). Community-acquired MRSA infections, once rare, have increased in frequency (*16*,*17*).

Clinicians are faced with the dilemma of attempting to limit broad-spectrum antimicrobial drug use on a population level while trying to maximize therapeutic success in individual patients (*18*). Routine prescribing of fluoroquinolones for CAP may limit the possibility of therapeutic failure due to drug-resistant organisms but may compromise the future effectiveness of this class of drugs. Practice guidelines available for management of CAP from various professional societies provide mixed messages on the use of fluoroquinolones, particularly for patients eligible for outpatient treatment. The American Thoracic Society recommends reserving fluoroquinolones for outpatients with cardiopulmonary disease or other modifying factors, advocating a macrolide or doxycycline for patients without such coexisting conditions (*19*). The Drug-Resistant *Streptococcus pneumoniae* Working Group recommends reserving fluoroquinolones for patients whose treatment has failed on other regimens or those with documented infections due to DRSP (*20*). Previous guidelines of the Infectious Diseases Society of America considered macrolides, doxycycline, or fluoroquinolones as equivalent options for treating outpatients, with the suggestion that older patients and those with underlying disease have a stronger indication for fluoroquinolone therapy (*21*). A recent update of these guidelines categorizes patients according to whether they recently received antimicrobial drugs and presence of underlying conditions: patients without underlying illnesses and no recent antimicrobial drug therapy should receive a macrolide or doxycycline, whereas fluoroquinolones are an option for the other groups (*22*). Such extensive subclassification and conflict among guidelines may pose difficulties for clinicians practicing in busy outpatient settings (*23*). Without clear and consistent guidelines, clinicians may base their therapeutic decisions on tradition, the practice of colleagues, or advice from pharmaceutical sales representatives, rather than the best evidence.

We examined a database of office visit and pharmacy claims from 4 large managed-care organizations in Colorado. Our objective was to determine the patterns of antimicrobial drug prescribing, especially fluoroquinolone use, in a group of outpatients with CAP without serious underlying conditions.

## Methods

We used administrative claims data from 4 healthcare organizations in Colorado. Identifiable patient information was removed before the information was provided, and patients were assigned a unique identification number for the purpose of data manipulation. Information contained in the database included the patient’s date of birth, sex, visit date, health plan, provider identification and specialty, up to 3 International Classification of Diseases, 9th edition, Clinical Modification (ICD-9-CM) diagnostic codes, and drugs prescribed during the visit. Data were available from March 1, 2000, to March 1, 2003. The study received approval from the institutional review board of the University of California, San Francisco.

Our criteria for inclusion in the study were age >18 years, primary diagnosis of CAP (based on ICD-9-CM codes 481, 482, 483, 485, and 486), and prescription of an antimicrobial agent associated with the visit. As serious coexisting conditions may justify the use of fluoroquinolones according to some guidelines, we excluded those patients with coexisting conditions to examine prescribing patterns in an otherwise healthy population. Specifically, we excluded patients who had a second or third diagnosis of chronic obstructive pulmonary disease, congestive heart failure, diabetes, lung cancer, renal failure, atrial fibrillation, respiratory failure, pleural effusion, Parkinson disease, multiple sclerosis, and asphyxia. We excluded patients who had sought treatment for an acute respiratory tract infection (bronchitis, pharyngitis, otitis media, sinusitis, and upper respiratory tract infection) or urinary tract infection during the 4 weeks before the visit for pneumonia. Consequently, we excluded results for the first month of the study (March 2000) since data regarding prior visits were unavailable for that group. Finally, we limited the dataset to 1 pneumonia visit per patient to reduce the likelihood of including patients whose previous therapy had been unsuccessful. We categorized patients by age into 3 strata: 18–44 years, 45–64 years, and >65 years. We also categorized patients according to health plan (1 through 4). For categorization by year, we used March 1 as the start and end date (e.g., year 2001 was March 1, 2001, to March 1, 2002).

Antimicrobial agents were identified by using National Drug Codes. Antimicrobial drugs were assigned to one of the following categories: tetracyclines (doxycycline, tetracycline), macrolides (azithromycin, clarithromycin, erythromycin), fluoroquinolones (ciprofloxacin, levofloxacin, ofloxacin, moxifloxacin, gatifloxacin), aminopenicillins (amoxicillin, amoxicillin/clavulanate), cephalosporins (primarily cefuroxime and cefprozil), sulfonamides (trimethoprim-sulfamethoxazole), and other. Only 1 antimicrobial agent was recorded per patient visit. We used the visit date to analyze data by year of prescription (2000, 2001, or 2002).

Comparisons between proportions in groups were performed by using the Mantel-Haenszel χ^2^ test for trend, with a significance level of 0.05. Logistic regression analysis was performed on the group of patients without coexisting conditions. The outcome was prescription of a fluoroquinolone. Variables included in the model were year of treatment, age (by category), patient sex, and health plan; and interactions between year of treatment with age, patient sex, and health plan were also tested. All analyses were performed by using SAS, version 8.2 (SAS, Cary, NC, USA).

## Results

Inclusion criteria were met by 5,001 patients in our database. Of our sample, 463 patients were excluded because of >1 conditions. Excluded patients were more likely to be in 1 of the older age categories (p < 0.001). Characteristics of the final group of study patients (N = 4,538) without coexisting conditions are presented in Table 1.

**Table 1:** Pneumonia patients with no serious underlying conditions treated with antimicrobial drugs

Characteristic	No. of patients (%) (N = 4,538)
Age (y) 18–44 45–64 >65	1,581 (35) 1,610 (35) 1,347 (30)
Sex Male Female	2,103 (46) 2,435 (54)
Study year 2000 2001 2002	1,827 (40) 1,328 (30) 1,383 (30)

**Figure 1:**
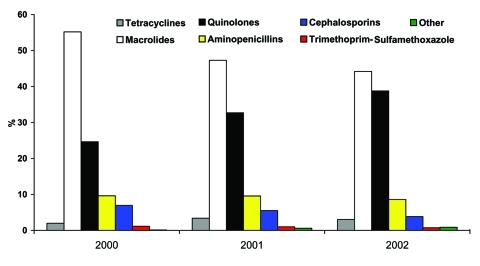
Antimicrobial drug treatment of outpatient pneumonia by year. Percentage of all study patients receiving a particular class of antimicrobial drug for an episode of community-acquired pneumonia for each year of the study, across all age groups.

Data from the 4,538 patients were analyzed to determine use of antimicrobial agents. Figure 1 shows changes in antimicrobial prescribing from 2000 to 2002 for all patients. Use of fluoroquinolones increased from 25% of all prescriptions in 2000 to 39% in 2002 (p < 0.001). Macrolide use decreased from 55% in 2000 to 44% in 2002 (p < 0.001). Aminopenicillin use (≈9%) did not change significantly, but cephalosporin use decreased by almost half (7% to 4%). Use of tetracyclines, sulfa drugs, and other antimicrobial agents was minimal. Of note, fluoroquinolone use in the 18– to 44-year age group more than doubled from 2000 to 2002 (14% to 30%)

**Figure 2:**
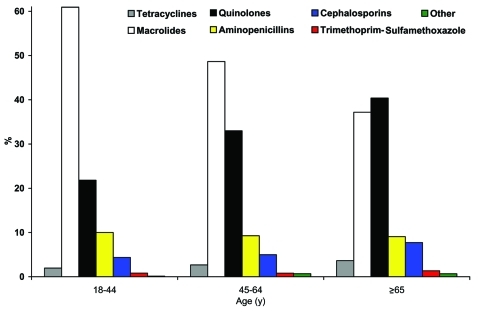
Antimicrobial drug treatment of outpatient pneumonia by age. Percentage of all study patients receiving a particular class of antimicrobial drug for an episode of community-acquired pneumonia by age group, across all study years.

Figure 2 shows the distribution of antimicrobial prescribing between the 3 age categories for all years in the study. Use of fluoroquinolones differed significantly between groups (p < 0.001); patients in the oldest group received more fluoroquinolones (40%) than those in the 45- to 64- (33%) and 18- to 44-year groups (22%). Macrolide use was highest in persons 18–44 years (61%), followed by those 44–65 years (49%) and persons >65 years (37%) (p < 0.001). Differences in prescribing of aminopenicillins, cephalosporins, and tetracyclines also were observed across age groups. Among fluoroquinolones prescribed over all years of the study, 74% were for levofloxacin; among all macrolides, 72% were for azithromycin.

Results of logistic regression analysis to determine predictors of fluoroquinolone use are presented in Table 2. When the 45- to 64-year age group was used as a referent, patients in the older group were more likely, and those in the younger group less likely, to receive fluoroquinolones. Likelihood of fluoroquinolone use also differed significantly by health plan: 1 health plan was less likely to prescribe fluoroquinolones. The interaction between year of prescription and age category was of marginal significance (p = 0.0855), which suggests that increases in fluoroquinolone use were similar across age groups. However, a significant interaction occurred between year of prescription and health plan (p < 0.0001), which indicates that changes in fluoroquinolone prescription rates differed by health plan.

**Table 2:** Logistic regression analysis of factors predicting fluoroquinolone use

N = 4,538	Odds ratio (95% CI)* for receipt of fluoroquinolone
Age (y) 18–44 45–64 >65	0.56 (0.48–0.66) 1 1.62 (1.38–1.91)
Year (later year)	1.40 (1.29–1.51)
Healthcare plan 1 2 3 4	1 0.97 (0.77–1.21) 0.80 (0.66–0.97) 1.23 (0.93–1.62)

*CI, confidence interval.

## Discussion

We found significant changes in the pattern of antimicrobial prescribing for the outpatient management of patients with CAP from 2000 to 2002. Fluoroquinolone use increased by >50%, from 25% to 39% of all prescriptions. This increase was at the expense of the macrolide class of antimicrobial drugs, the use of which declined 20% during the study period. Use of β-lactam drugs and doxycycline was low throughout the study period. Although fluoroquinolones were prescribed for older patients more frequently than for younger patients, the growth in fluoroquinolone use over the study was similar across all age groups.

Few published studies have documented trends in use of fluoroquinolones for the management of respiratory tract infections in the community. No study has specifically addressed the use of fluoroquinolones for pneumonia. In a national sample of U.S. office-based physicians from 1992 to 2000, a 78% increase in the use of fluoroquinolones across all indications was documented (*24*). Chen et al. noted a 5-fold increase in community fluoroquinolone prescribing for all indications in Canada from 1988 to 1997 (*5*). In a survey of U.S. community-based prescribing from 1991 to 1999, use of fluoroquinolones for upper respiratory tract infections in adults increased from <1% to 13% of antimicrobial drug prescriptions (*25*).

A number of factors may contribute to the observed increased fluoroquinolone use, including the convenience of once-daily dosing, reliable spectrum of activity against CAP pathogens, and relatively low toxicity. In addition, changes in health plan formularies, pharmaceutical advertising, and concerns about resistance to standard therapies may have influenced these prescribing trends*.*

We found age-specific differences among patients for whom fluoroquinolones were prescribed. Patients in the older age groups were more likely to receive fluoroquinolones than those in persons 18–44 years, even after those with underlying conditions were excluded. Age itself may be an important underlying condition as well as a risk factor for drug-resistant *S. pneumoniae* (*26*). Thus, clinicians may choose to use drugs they perceive to be more potent to reduce the risk for treatment failure in this population. In a survey of fluoroquinolone use from the National Hospital Ambulatory Medical Care Survey from 1993 to 1998, persons >65 years of age had the highest use of fluoroquinolones (12.4 prescriptions/100 persons/year, compared to a mean of 4.6/100 persons/year for all groups) (*27*).

A recent study evaluated outpatient fluoroquinolone use for CAP in 6 emergency departments in Canada (*28*). The most commonly prescribed antimicrobial drugs were macrolides (53%) and fluoroquinolones (32%; 98% of these prescriptions were for levofloxacin). Similar to our results in this study, increasing age was a significant predictor of levofloxacin use; 25% of patients 16–40 years of age received levofloxacin, compared to 28% of those 41–64 years and 47% of those ≥65 years. We also determined appropriateness of levofloxacin use on the basis of Canadian guidelines. When we used an interpretation of these guidelines in which fluoroquinolone use in all patients with chronic obstructive pulmonary disease or recent antimicrobial drug use was considered appropriate, 51% of levofloxacin prescriptions were considered inappropriate.

We did not have adequate information to fully assess appropriateness of therapy, and the lack of agreement among currently published U.S. guidelines prevents establishing a universal benchmark. However, in the absence of coexisting conditions, the prognosis for patients <45 years of age with CAP is favorable. The American Thoracic Society, as well as CDC, recommends use of either a macrolide or doxycycline in this group (current Infectious Diseases Society of America guidelines would use a fluoroquinolone if the patient had recently used an antimicrobial agent). Although fewer fluoroquinolones were used in the younger age groups, substantial use was noted in all age groups, and increases in the rate of fluoroquinolone use were independent of age group. These findings suggest that those prescribing antimicrobial drugs may be increasingly using fluoroquinolones as a “one-size-fits-all” regimen without accounting for differences due to age and other risk factors. Clarifying the appropriateness of fluoroquinolone use on the basis of patient characteristics should be a goal of future joint guidelines for CAP. We also observed that patterns of fluoroquinolone prescribing varied across health plans. Although this finding probably reflects differences in each health plan’s pharmacy benefits management programs, our study did not collect information to help further characterize this finding. Further investigation of the differences between these programs is warranted because these studies might identify or inform effective interventions to reduce excess fluoroquinolone prescribing.

Our study has a number of limitations. The cohort consists of patients seen in a particular geographic area. Antimicrobial drug use may vary significantly by region. The dataset consisted of patients enrolled in managed-care organization healthcare plans. Prescribing patterns for these patients may differ from those for other populations. The database captured only the first 3 ICD-9 codes associated with a patient visit, which we used to exclude patients with notable underlying conditions. Some coexisting conditions may have been coded as 4th or 5th diagnoses, or not coded at all, in which case our sample would not have excluded all patients with these coexisting conditions as we planned. We did not measure outcomes, such as return visits or deaths, to assess the effectiveness of the prescribed regimens. We were not able to exclude all patients with recent antimicrobial drug therapy. However, we did exclude patients with a recent visit for an acute respiratory infection or urinary tract infection, many of whom would have received an antimicrobial drug. We also did not have data on local antimicrobial resistance patterns to assess appropriateness of empiric therapy or changes in resistance over the course of the study.

In summary, our study demonstrates an increase in fluoroquinolone use to treat outpatient CAP among a cohort without complicating coexisting conditions. This increasing use of fluoroquinolones, especially in otherwise healthy patients whose infections are not likely to fail to respond to treatment or whose infections are not likely to be caused by resistant organisms, may threaten the future effectiveness of this drug class. Harmonization of expert guidelines regarding the role of fluoroquinolones in the outpatient management of CAP is recommended.
